# The effect of selected aminoglycoside antibiotics on human serum albumin antioxidant activity: a spectroscopic and calorimetric comparative study

**DOI:** 10.1007/s43440-023-00529-6

**Published:** 2023-09-13

**Authors:** Wojciech Rogóż, Kinga Mac, Aleksandra Owczarzy, Karolina Kulig, Jadwiga Pożycka, Małgorzata Maciążek-Jurczyk

**Affiliations:** Department of Physical Pharmacy, Faculty of Pharmaceutical Sciences in Sosnowiec, Medical University of Silesia, 40-055 Katowice, Poland

**Keywords:** Aminoglycosides, Albumin, Calorimetry, Spectroscopy, Antioxidants

## Abstract

**Background:**

Human serum albumin (HSA) is a valuable component of non-enzymatic and endogenous antioxidant mechanisms. The antioxidant activity of HSA can be modulated by ligands, including drugs. Although this is a central topic in the field of oxidation, there is still a lack of information about the protection against the effects of elevated free radical levels.

**Methods:**

The aim of this study was to investigate the antioxidant activity of kanamycin (KAN) and neomycin (NEO) and their effect on the antioxidant potential of HSA using spectroscopic and microcalorimetric techniques.

**Results:**

Despite the fact that kanamycin and neomycin interact with HSA, no changes in the secondary structure of the protein have been observed. The analysis of the aminoglycoside antibiotics showed their low antioxidant activity and a synergistic effect of the interaction, probably due to the influence of ligands (KAN, NEO) on the availability of HSA amino acid residues functional groups, such as the free thiol group (Cys-34).

**Conclusions:**

Based on the spectroscopic and microcalorimetric data, both KAN and NEO can be considered modulators of the HSA antioxidant activity.

## Introduction

Free radicals are molecules with unpaired electron(s) on the valence shell [1–3] that can be either harmful or helpful to the human body [1, 2]. They are responsible for the maintenance of the redox balance as well as the activation of various signaling pathways. On the other hand, the high reactivity of free radicals causes numerous adverse effects at the molecular, tissue, and organ levels. They can disrupt normal metabolic pathways and damage the building process of proteins, nucleic acids, lipids, and other micro- and macromolecules [1–3]. Increased levels of free radicals in the human body are often accompanied by the developing of inflammation, which is a common feature of numerous diseases [2]. Restoring the homeostasis is a long-term process that is in particular controlled by endogenous antioxidant mechanisms [3]. The major endogenous antioxidants include catalase or superoxide dismutase, as well as non-enzymatic agents, such as glutathione, bilirubin, coenzyme Q10, uric acid, metallothioneins, or melatonin [4, 5].

Human serum albumin (HSA), as the most abundant protein found in human plasma, plays a key role in the non-enzymatic, endogenous antioxidant mechanisms [3, 6–11]. HSA can bind both exogenous and endogenous ligands, such as drugs, toxins, metal ions, thyroxine, heme, bilirubin, nucleic acids, and fatty acids [6, 11]. The main two HAS bindings include Sudlow’s binding site I (subdomain IIA) and site II (subdomain IIIA) [6–8]. It is worth noting that the formation of protein–ligand complexes in binding sites induces various conformational changes [8]. Furthermore, HSA maintains osmotic pressure and is also viewed as an important antioxidant [7, 8, 11]. The role of HSA as an antioxidant is associated with the presence of a single, free thiol group (Cys-34) of amino acid residues. Methionine residues, such as Met-87, Met-123, Met-298, Met-329, Met-446, and Met-548, are also crucial in forming the antioxidant potential of HSA [7, 9–11].

To summarize, to evaluate the activity of antioxidants, it is necessary to analyze chemical reactions they are involved in, including mutual interactions and reactions with endogenous factors such as proteins [9, 12]. The interaction between antioxidants can be synergistic (s), antagonistic (an), or additive (ad) [13, 14]. Both endogenous and exogenous compounds can modulate the HSA ability to scavenge free radicals through endogenous, non-enzymatic antioxidant mechanisms [12]. Among the numerous exogenous modulators of the HSA antioxidant activity, there are non-steroidal anti-inflammatory drugs [15], losartan and furosemide [16], as well as alkaloids like quinine [17].

Aminoglycosides are a widely used group of antibiotics. Due to the broad spectrum of antibacterial activity, rapid bactericidal speed, and the possibility of chemical modification to amphiphilic aminoglycosides, they are important from the pharmacology research perspective [18, 19]. Aminoglycosides are antibacterial agents with a broad spectrum of actions that disrupt protein translation on prokaryotic ribosomes (70S) [18, 20, 21] and are used for the treatment of infections caused by the *Enterobacteriaceae* family, as well as *Francisella tularensis*, *Yersinia pestis*, *Staphylococcus aureus*, and *Pseudomonas aeruginosa* [18–20]. Nevertheless, aminoglycosides are drugs with a narrow therapeutic window and they cause severe and often irreversible side effects, especially nephrotoxicity and ototoxicity [21, 22].

The main aim of the study was to evaluate the influence of aminoglycoside antibiotics such as kanamycin (KAN) and neomycin (NEO) on human serum albumin (HSA) antioxidant potential based on the comprehensive spectroscopic and microcalorimetric analysis of HSA-ligands binding and protein secondary structure.

## Materials and methods

### Chemicals

**Fig. 1:**
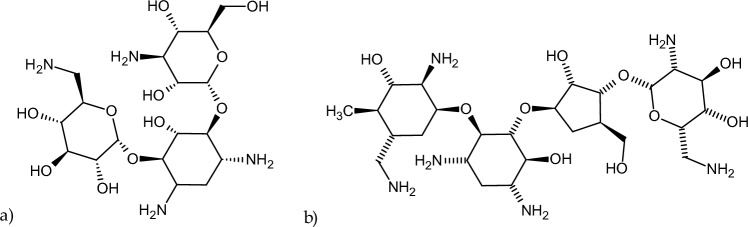
The structural formula of **a** kanamycin (*KAN*) and **b** neomycin (*NEO*) (ChemSketch 12.1.0.31258)

Human serum albumin (HSA) fraction V (Lot No. 4971 K) was obtained from MP Biomedicals. Kanamycin (KAN, Fig. 1a; Lot No. 48H0582) as well as 2,2′-azino-bis(3-ethylbenzothiazoline-6-sulfonic acid) diammonium salt (ABTS; Lot No. SLBZ8095), 2,2-diphenyl-1-picrylhydrazyl (DPPH; Lot No. STBH727) were obtained from Sigma-Aldrich, while neomycin sulfate (NEO, Fig. 1b; Series No. 010209) from PPH Galfarm Sp. z o. o. Potassium persulfate (K_2_S_2_O_8_) and ascorbic acid (C_6_H_8_O_6_) were purchased from Chempur. Dipotassium hydrogen phosphate pure p.a. (K_2_HPO_4_) and sodium dihydrogen phosphate (NaH_2_PO_4_) were from Eurochem BGD Sp. z o. o. All chemicals were of analytical grade and used without further purification.

### Methods

#### Circular dichroism (CD)

To investigate changes in the secondary structure of HSA under the influence of ligand binding, the JASCO J-1500 Spectropolarimeter (JASCO International Co., Ltd., Hachioji, Tokyo, Japan) was used. All analyzed solutions were prepared with the use of phosphate buffer (0.05 M, pH 7.4). The concentration of HSA was 3 × 10^–6^ M, while the concentration of ligand stock solution was 4.13 × 10^–4^ M. The ligand:HSA molar ratio was 2:1. All measurements were determined using quartz cuvettes with a 1 mm path length. The spectra were obtained at wavelengths in the range between 200 and 250 nm, at 0.5 nm intervals, and at 50 nm/min scanning speed. A thermostatic Peltier cell holder temperature was maintained constant at 298 ± 0.05 K.

The mean residue ellipticity [Θ]_MRW_ was calculated using Eq. (1) [23, 24]

1 $$\left[ \Theta \right]_{{{\text{MRW}}}} = \frac{{{\text{MRW}} \times \Theta }}{{10 \times {\text{l}} \times {\text{m}}}}\left[ {{\text{deg }} \times {\text{ cm}}^{{2}} \times {\text{ dmol}}^{{ - {1}}} } \right],$$

where Θ is the observed ellipticity for a particular wavelength [deg]; m is concentration of the protein [g × cm^−3^]; 1 is the pathlength [cm]; MRW is a mean residue weight (MRW_HSA_ = 113.7 [Da]).

#### Nano-isothermal titration calorimetry (nanoITC)

The nanoITC experiments were performed at 298 K with a nano-isothermal titration calorimeter (TA Instruments, New Castle, USA). All samples were degassed (degassing time *t* = 15 min) using Degassing Station (TA Instruments, New Castle, USA). Calorimetric measurements were determined based on the experimental and theoretical parameters [15, 16, 25–28] (Table 1). All solutions were prepared using a phosphate buffer (0.05 M, pH 7.4).

**Table 1:** Experimental dataset

Property	Value
Syringe concentration (10^–3^ mol/dm^3^)	1.2
Cell concentration (10^–5^ mol/dm^3^)	3
Initial cell Vol (μl)	300
Inj. interval (s)	180
Inj. volume (μl)	2.38
Temperature (K)	298
Stir rate (RPM)	300

The Gibbs free energy change *ΔG* has been obtained based on Eq. (2) [29]

2 $$\Delta {\text{G }} = \, \Delta {\text{H }} - {\text{ T}}\Delta {\text{S }}\left[ {{\text{kcal }} \times {\text{ mol}}^{{ - {1}}} } \right],$$

where ΔH is enthalpy change [kcal × mol^−1^]; *T *is temperature [K]; ΔS is entropy change [kcal × mol^−1^ K^−1^].

#### UV–Vis (antioxidant activity: DPPH and ABTS assays)

Spectrophotometric measurements were performed using JASCO V-730 UV–visible spectrophotometer (JASCO International Co., Ltd., Hachioji, Tokyo, Japan). Samples of HSA, KAN, and NEO solutions were prepared using a phosphate buffer (0.05 M, pH 7.4). The DPPH and ABTS assays were conducted according to the modified protocols of Rogóż et al. [15–17].

To measure the antioxidant activity under denaturing conditions, a DPPH assay was conducted. The sample concentrations were 2 × 10^–4^ M and 4 × 10^–4^ M, for HSA and ligands (KAN and NEO), respectively. The protein and ligand mixture was prepared at HSA:ligand 1:1 v:v and 1:2 molar ratios. The working reagent was prepared by dissolving DPPH in 96% ethanol (1 × 10^–4^ M concentration) and mixed with samples at DPPH:sample 1:1 v:v ratio. The absorbance was measured at the wavelength of *λ*_max_ 517 nm and observed at 5, 15, 30, 45, and 60 min after the initiation of the radical reaction.

To measure the antioxidant activity in non-denaturing conditions, the ABTS assay was performed. The samples concentrations were 1 × 10^–5^ M and 2 × 10^–5^ M, for HSA and ligands, respectively. The protein and ligand mixture was prepared at HSA:ligand 1:1 v:v and 1:2 molar ratios. The working reagent was prepared using ABTS and potassium persulfate solutions at 5 × 10^–3^ M and 1.74 × 10^–3^ M concentrations, respectively. All samples were placed in the dark for 16 h and then diluted to obtain an absorbance of 1 at λ_max_ 734 nm. To initiate the radical reaction, the samples were mixed with the working reagent ABTS:sample 1:1 v:v ratio. The absorbance was measured at a wavelength of λ_max_ 734 nm and observed at 5, 15, 30, 45, and 60 min after the initiation of the radical reaction.

All samples were mixed with Vortex (IKA Vortex Genius 3), stored, and measured at 298 K temperature.

The % inhibition has been obtained based on Eq. (3) [30, 31]

3 $$\% {\text{ inhibition }} = \left( {\frac{{A_{0} - A_{1} }}{{A_{0} }}} \right) \times { 1}00\% ,$$

where *A*_*0*_, *A*_*1*_ are the absorbance of DPPH or ABTS in the absence and presence of the samples, respectively.

The % inhibition values were converted to ascorbic acid equivalent antioxidant capacity (AAEAC). The calibration curves of ascorbic acid (AA) at concentrations ranged from 1.20 × 10^–6^ M to 7.67 × 10^–5^ M (DPPH assay) and from 9.58 × 10^–7^ M to 1.53 × 10^–5^ M (ABTS assay) were prepared.

To determine the effect of macromolecules and ligands on the complex formation, expected versus designated values of antioxidant activity have been presented. The AAEAC of the mixtures was calculated based on the AAEAC values obtained for HSA and the particular component. The effect of solution dilution has been taken into account. The methodology was developed on the basis of Guimarães et al.’s [14] work.

#### UV–Vis (ligand–HSA interaction)

Absorption spectra of KAN, NEO, HSA, (KAN-HSA)_complex_, and (NEO-HSA)_complex_ were performed to study the interaction between ligands and HSA. Spectrophotometric measurements were obtained using JASCO V-730 (JASCO International Co., Ltd., Hachioji, Tokyo, Japan) at wavelengths in the range between 230 and 400 nm, at 0.2 nm intervals, 40 nm/min scanning speed, and 0.24 s response. The samples were prepared in a phosphate buffer (0.05 M, pH 7.4). The concentration of HSA was 2 × 10^–5^ M, while the concentration of ligands was 4 × 10^–5^ M. Based on the absorption spectra of HSA, (KAN-HSA)_complex_, and (NEO-HSA)_complex_, the second derivative of differential absorption spectra was determined (algorithm: Savitzky-Golay; data points: 25). The r parameter was calculated on the basis of Eq. (4) [32–34]

4 $$r=\frac{a}{b},$$

where *a*, *b* are the values of two adjacent peaks (*a* = a_1_–a_2_; *b* = b_1_–b_2_) selected from among the peaks recorded for two wavelength ranges (250–270 nm and 270–310 nm) [32–34].

### Statistics

All results were presented as a mean ± relative standard deviations (mean ± SD) and statistical significance was assessed. All measurements were made in at least three independent experiments. Based on the Shapiro–Wilk test, a 2D histogram, a Q–Q plot, and a box plot, the obtained data had a normal or very close-to-normal distribution. The equality of variance has been calculated using Levene's test and presented for the CD, DPPH, and ABTS measurement results while for the interaction, homogeneity of variance could not be confirmed. Therefore, one-way ANOVA test or one-way ANOVA for repeated measures with Tuckey`s post hoc test (for CD, DPPH, and ABTS measurement results) and Kruskal–Wallis one-way analysis of variance test (for interaction analysis) were used to confirm differences between the obtained mean/median (confidence level: *p* < 0.05). The results were analyzed using the following software: OriginPro software version 8.5 SR1 (Northampton, MA, USA), Statistica (data analysis software system) version 13, Spectra Manager Version 2.13.00 2002–2015 (JASCO International CO., LTD., Hachioji, Tokyo, Japan), and Launch NanoAnalyze program (TA Instruments, New Castle, USA).

## Results

### Spectropolarimetric measurements of the impact of KAN and NEO on HSA secondary structure

**Fig. 2:**
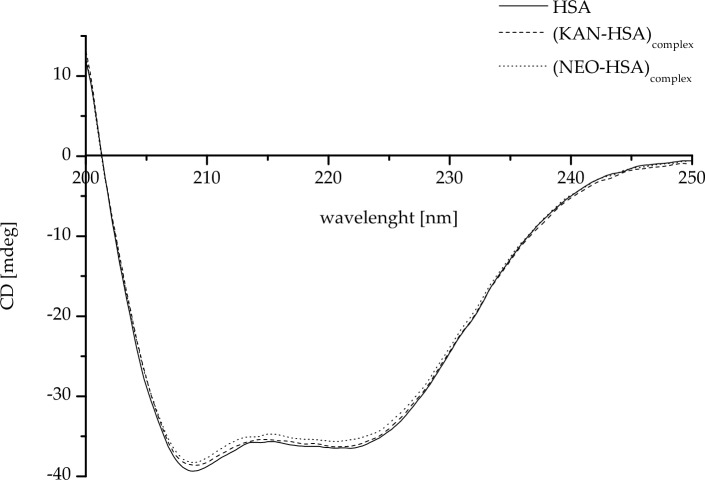
Circular dichroism (CD) spectra of human serum albumin, in the absence (HSA) and presence of kanamycin (KAN-HSA)_complex_ and neomycin NEO-HSA)_complex_

To analyze the impact of KAN and NEO on HSA`s secondary structure, far-UV CD spectra were conducted. The CD spectra, the mean residue ellipticity for (KAN-HSA)_complex_, and (NEO-HSA)_complex_ as well as the percentage (%) content of the secondary structure elements of HSA (Young’s reference model) in the absence and presence of the analyzed drugs are shown in Fig. 2 and collected in Tables 2 and 3, respectively.

**Table 2:** The values of the mean residue ellipticity [Θ_MRW_] of human serum albumin, in the absence (HSA) and presence of kanamycin (KAN-HSA)_complex_ and neomycin (NEO-HSA)_complex_)

Samples	λ_min_	[Θ_MRW_] ± SD* [deg × cm^2^ × dmol^−1^]
HSA	208.8	– 22,607.76 ± 396.38
	221.4	– 20,981.82 ± 330.66
(KAN-HSA)_complex_	208.8	– 21,986.84 ± 55.95
	221.4	– 20,364.08 ± 230.67
(NEO-HSA)_complex_	208.8	– 21,859.54 ± 179.25
	221.4	– 19,140.73 ± 114.11

*SD standard deviation; *n* = 3

**Table 3:** Human serum albumin’s secondary structure elements content (%) in the absence and presence of kanamycin and neomycin, respectively

Samples	% α-Helix ± SD*	% β-sheet ± SD*	% Turn ± SD*	% Other ± SD*
HSA	34.33 ± 0.47	12.60 ± 0.70	21.07 ± 0.31	32.03 ± 0.12
(KAN-HSA)_complex_	35.20 ± 0.60	11.33 ± 0.49	21.47 ± 0.12	32.03 ± 0.21
(NEO-HSA)_complex_	35.07 ± 0.38	11.77 ± 0.55	21.30 ± 0.30	32.07 ± 0.46
One-way ANOVA	*F*_2_ = 2.70 *p* = 0.15	*F*_2_ = 3.60 *p* = 0.09	*F*_2_ = 1.85 *p* = 0.24	*F*_2_ = 0.0 *p* = 0.99

[ligand]:[HSA] 2:1 molar ratio; Young's reference model; *SD standard deviation; *n* = 3

The CD spectrum of HSA is characterized by a strong double minimum at λ_min_ 208.8 nm and λ_min_ 221.4 nm, indicating that α-helix is the dominant secondary structure (Fig. 2). The formation of HSA complexes with the ligands did not significantly affect the changes in either the wavelength at which the spectrum reaches its minima or the mean residue ellipticity values (Table 2).

As indicated in Table 3, the binding of KAN and NEO to HSA did not show significant changes beyond 1% in the secondary structure of HSA. The observed slight increase in the percentage of α-helix was not significant (Table 3). The protein retained a constant level of α-helix structure (approximately 34.5%), as did the proportions of each type of secondary structure (α-helix:β-sheet:turn:other 3:1:2:3).

### Spectrophotometric analysis of the interaction between HSA and ligands

**Fig. 3:**
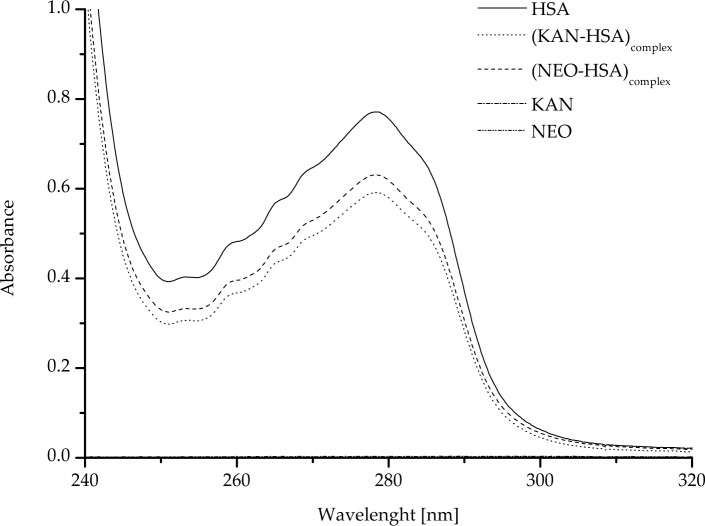
The absorption spectra of human serum albumin (HSA), kanamycin (KAN), neomycin (NEO), as well as ligand–protein complexes: (KAN-HSA)_complex_ and (NEO-HSA)_complex_ ([HSA] = 2 × 10^–5^ M, [KAN] = [NEO] = 4 × 10^–5^ M)

The absorption spectra of HSA and analyzed ligands (KAN, NEO) as well as (KAN-HSA)_complex_ and (NEO-HSA)_complex_ are presented in Fig. 3 and collected in Table 4.

**Table 4:** The mean absorbance values for human serum albumin (HSA), kanamycin (KAN), neomycin (NEO), as well as ligand–protein complexes: (KAN-HSA)_complex_ and (NEO-HSA)_complex_ ([HSA] = 2 × 10^–5^ M, [KAN] = [NEO] = 4 × 10^–5^ M)

Wavelength λ [nm]		Absorbance ± SD*				Effect ***(I/NI)
	HSA	KAN	NEO	(KAN-HSA)_complex_	(NEO-HSA)_complex_	
251.0	0.393 ± 0.004	N/S**	N/S**	0.298 ± 0.001	0.325 ± 0.013	I
260.2	0.483 ± 0.004	N/S**	N/S**	0.368 ± 0.001	0.396 ± 0.015	I
265.0	0.567 ± 0.004	N/S**	N/S**	0.433 ± 0.000	0.465 ± 0.017	I
269.4	0.641 ± 0.004	N/S**	N/S**	0.491 ± 0.001	0.525 ± 0.019	I
278.4	0.771 ± 0.004	N/S**	N/S**	0.591 ± 0.000	0.631 ± 0.022	I

^*^SD standard deviation; **N/S no signal; ***I interaction (the absorbance for ligand–protein complexes differs by more than 5% from the absorbance for HSA), *NI* no interaction; *n* = 3

As can be seen from the data presented in Fig. 3 and collected in Table 4, the absorption spectra of HSA, (KAN-HSA)_complex_, and (NEO-HSA)_complex_ do not overlap. In the analyzed wavelength range, both KAN and NEO do not absorb. The shape of the absorption spectra of HSA, (KAN-HSA)_complex,_ and (NEO-HSA)_complex_ did not change, but the absorbance decreased under the influence of both KAN and NEO.

**Fig. 4:**
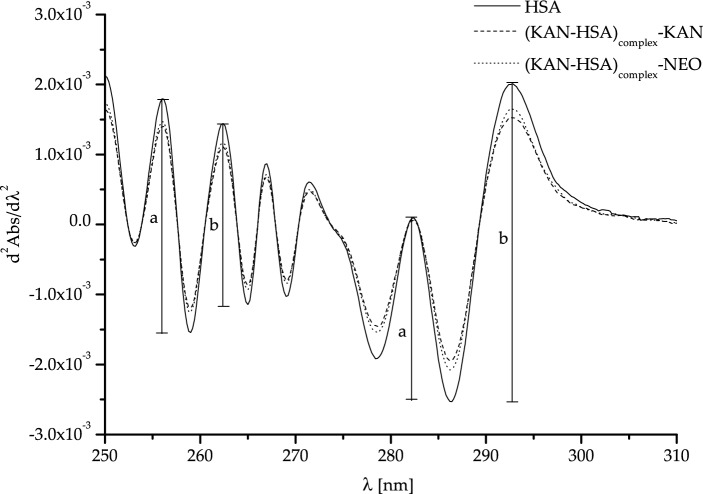
Second derivative of differential absorption spectra of human serum albumin (HSA), as well as ligand–protein complexes: (KAN-HSA)_complex_ and (NEO-HSA)_complex_ ([HSA] = 2 × 10^–5^ M, [KAN] = [NEO] = 4 × 10^–5^ M); a, b the values of two adjacent peaks (a = a_1_–a_2_; b = b_1_–b_2_)

Based on the second derivative of differential absorption spectra (Fig. 4) and Eq. (4), the median *r* values were calculated and are collected in Table 5.

**Table 5:** The median r values determined on the basis of the second derivative of differential absorption spectra of human serum albumin (HSA), as well as ligand–protein complexes: (KAN-HSA)_complex_ and (NEO-HSA)_complex_ ([HSA] = 2 × 10^–5^ M, [KAN] = [NEO] = 4 × 10^–5^ M); *λ* wavelength; *n* = 3

	*r* = $\frac{a}{b}$	
	λ 250–270 [nm] Me (1st quartile–3rd quartile)	λ > 270 [nm] Me (1st quartile–3rd quartile)
HSA	0.7059 (0.7041 – 0.7060) ^(a)^	0.4409 (0.4404 – 0.4455)
(KAN-HSA)_complex_	0.7284 (0.7261 – 0.7453) ^(a)^	0.4366 (0.4321 – 0.4381)
(NEO-HSA)_complex_	0.7197 (0.7089 – 0.7202)	0.4358 (0.4358 – 0.4366)
Kruskal–Wallis test	*H*_2_ = 7.20 *p* = 0.03	*H*_2_ = 5.42 *p* = 0.07

^(a)^statistically significant difference in “*λ* 250–270 nm” between ^(a)^ HSA vs. (KAN-HSA)_complex_

Based on the data presented in Fig. 4 and collected in Table 5, it can be concluded that in the wavelength range from 270 to 310 nm (λ > 270 nm) slight differences in the values of the r parameter calculated for native HSA and the protein under the influence of ligands were observed. On the other hand, in the range between 250 and 270 nm, statistically significant differences (based on the Kruskal–Wallis one-way test: *p* = 0.03) in the value of *r* parameter for HSA and (KAN-HSA)_complex_ [*p* value for multiple comparisons (two-sided), Kruskal–Wallis one-way test: *p* = 0.02] have been observed. The obtained results did not confirm (Kruskal–Wallis one-way test: *p* = 0.07) the differences between the *r* parameter calculated for HSA and (NEO-HSA)_complex_ (Fig. 4, Table 5).

### Calorimetric analysis of the HSA interaction with KAN and NEO

**Fig. 5:**
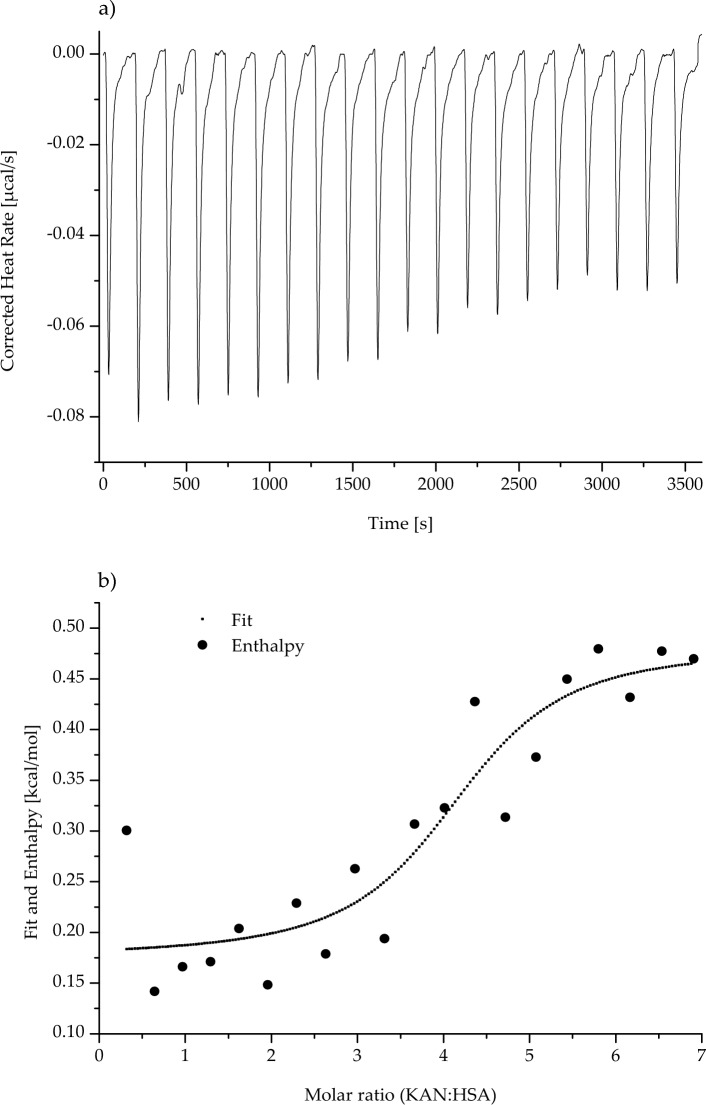
nanoITC (nano-Isothermal Titration Calorimetry) thermogram of human serum albumin (HSA) in the presence of kanamycin (KAN; KAN: HSA ~ 0.32:1 ÷  ~ 6.34:1 molar ratio); **a** shows the corrected raw heat data obtained from the consecutive injections, in turn, **b** presents the binding isotherm created by plotting areas of the heat peak in relation to the molar ratio of KAN to HSA. The lines present the best fit of the models used. *T* = 298 K

**Fig. 6:**
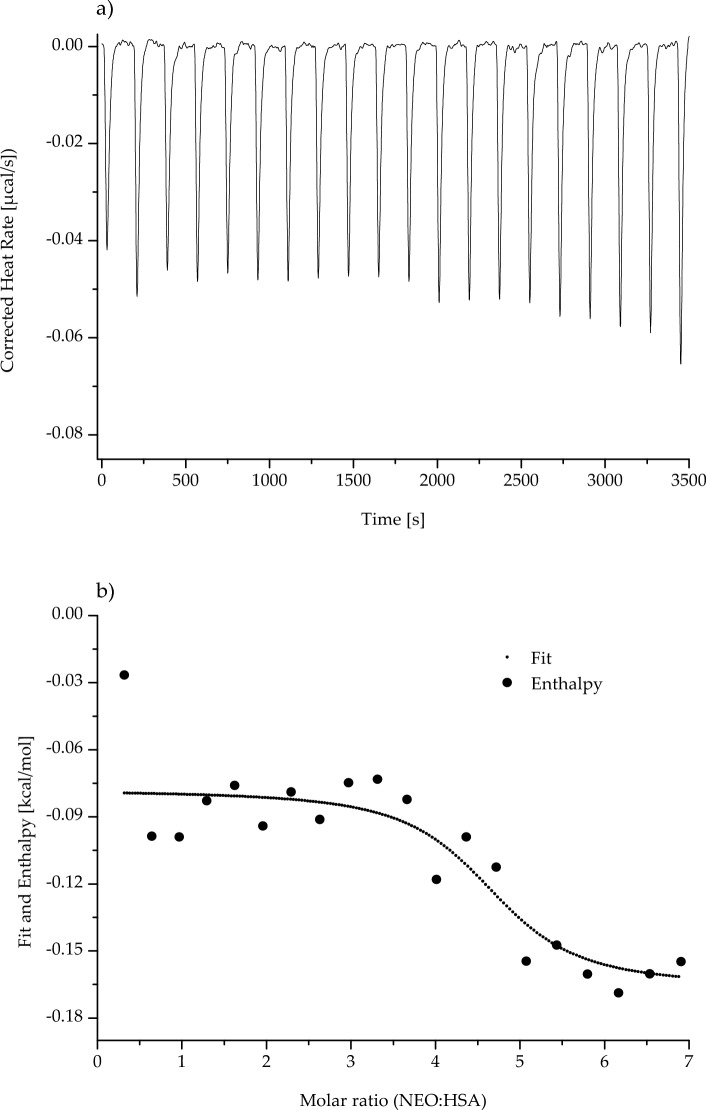
nanoITC (nano-Isothermal Titration Calorimetry) thermogram of human serum albumin (HSA) in the presence of neomycin (NEO; NEO:HSA ~ 0.32:1 ÷  ~ 6.34:1 molar ratio); **a** shows the corrected raw heat data obtained from the consecutive injections, whereas **b** presents the binding isotherm created by plotting areas of the heat peak in relation to the molar ratio of NEO to HSA. The lines present the best fit of the models used. *T* = 298 K

The interaction in KAN-HSA and NEO-HSA complexes were determined using microcalorimetric measurements. Thermograms of HSA in the presence of KAN and NEO are shown in Figs. 5 and 6, respectively. Binding parameters of drug–albumin interaction are collected in Table 6.

**Table 6:** Parameters (association constant, K_a_; stoichiometry, n; enthalpy change, ΔH; entropy change, ΔS, Gibbs free energy change, ΔG) of drugs-protein (kanamycin, KAN; neomycin, NEO; Human Serum Albumin, HSA) interaction [(KAN-HSA)_complex_, (NEO-HSA)_complex_]

Parameters ± SD*	(KAN-HSA)_complex_	(NEO-HSA)_complex_
*K*_a_ [dm^3^/mol]	(3.35 ± 0.19) × 10^5^	(9.57 ± 0.22) × 10^4^
n	3.57 ± 0.46	5.26 ± 1.71
ΔH [kcal/mol]	− 0.29 ± 0.03	0.21 ± 0.11
ΔS [cal/molK]	24.10 ± 1.11	24.67 ± 1.66
ΔG [kcal/mol]	− 7.39 ± 0.25	− 7.32 ± 0.73

^*^SD standard deviation; *n* = 3

Analyzing data collected in Table 6, the association constant (K_a_) obtained for the KAN and HSA system was higher than the K_a_ obtained for the (NEO-HSA)_complex_. The stoichiometry of reactions of both ligands with HSA was similar. About 4 KAN molecules and about 6 NEO molecules can interact with a single HSA molecule. Data presented in Figs. 5 and 6 and collected in Table 6 suggest that the process of (KAN-HSA)_complex_ formation was characterized by a negative value of enthalpy change (ΔH), while the process of (NEO-HSA)_complex_ formation was distinguished by a positive value of enthalpy change. The shape of the isotherm created by plotting areas of the heat peak in relation to the molar ratio of KAN to HSA was upward (Fig. 5), whereas the shape of the isotherm created by plotting areas of the heat peak in relation to the molar ratio of NEO to HSA was downward (Fig. 6). In both analyzed ligand–protein reactions, the entropy change (ΔS), as well as Gibbs free energy change (ΔG), were positive and negative, respectively (Table 6).

### Spectroscopic analysis of HSA, KAN, NEO, and their mixtures ability to scavenge free radicals in denaturing (DPPH assay) and non-denaturing (ABTS assay) conditions

UV–Vis spectroscopy is a valuable tool that allows the analysis of the antioxidant activity and the impact of selected ligands on the antioxidant activity of a protein. Based on the obtained absorbance values of individual samples, the antioxidant activity AAEAC values were calculated. Average AAEAC values for DPPH assay and ABTS assay are collected in Tables 7 and 8. Expected and designated values of antioxidant activity AAEAC of (KAN-HSA)_complex_, (NEO-HSA)_complex_ mixtures (DPPH and ABTS assays, respectively) are presented in Tables 9 and 10, respectively.

**Table 7:** The values of Ascorbic Acid Equivalent Antioxidant Capacity (AAEAC) at ligand:protein (human serum albumin *HSA*; kanamycin *KAN;* neomycin *NEO*) 2:1 molar ratio; DPPH assay

Antioxidant activity (AAEAC) ± SD* [µM AA] DPPH assay					
Samples	Time [min]				
	5	15	30	45	60
HSA	26.29 ± 1.65	34.11 ± 3.29 ^(a)^	36.78 ± 3.61 ^(c)^	35.68 ± 4.68 ^(e)^	34.2 ± 4.91 ^(g)^
KAN	10.69 ± 1.38	9.69 ± 0.56	N/A**	6.62 ± 1.30	8.17 ± 1.78
NEO	N/A**	N/A**	N/A**	N/A**	N/A**
(KAN-HSA)_complex_	27.73 ± 1.07	30.63 ± 1.1 ^(b)^	32.74 ± 7.41 ^(d)^	31.59 ± 5.26 ^(f)^	33.11 ± 4.79 ^(h)^
(NEO-HSA)_complex_	18.51 ± 2.31	13.44 ± 1.72 ^(a) (b)^	15.53 ± 7.16 ^(c) (d)^	14.64 ± 6.11 ^(e) (f)^	13.37 ± 5.87 ^(g) (h)^
One-way ANOVA for repeated measures	F_8_ = 2.41 *p* = 0.03				

Tuckey`s post hoc test: statistically significant difference between ^(a)^ HSA vs. (NEO-HSA)_complex_ for 15 min; ^(b)^ (KAN-HSA)_complex_ vs. (NEO-HSA)_complex_ for 15 min; ^(c)^ HSA vs. (NEO-HSA)_complex_ for 30 min; ^(d)^ (KAN-HSA)_complex_ vs. (NEO-HSA)_complex_ for 30 min; ^(e)^ HSA vs. (NEO-HSA)_complex_ for 45 min; ^(f)^ (KAN-HSA)_complex_ vs. (NEO-HSA)_complex_ for 45 min; ^(g)^ HSA vs. (NEO-HSA)_complex_ for 60 min; ^(h)^ (KAN-HSA)_complex_ vs. (NEO-HSA)_complex_ for 60 min

*SD standard deviation, **N/A not applicable); *n* = 4–6

**Table 8:** The values of Ascorbic Acid Equivalent Antioxidant Capacity (AAEAC) at ligand:protein (human serum albumin, HSA; kanamycin, KAN; neomycin, NEO) 2:1 molar ratio

Antioxidant activity (AAEAC) ± SD* [µM AA] ABTS assay					
Samples	Time [min]				
	5	15	30	45	60
HSA	2.56 ± 0.78	6.49 ± 0.62	8.43 ± 0.68	10.16 ± 0.68	12.49 ± 1.45 ^(a)^
KAN	0.84 ± 0.49	1.06 ± 0.60	1.54 ± 0.40	1.64 ± 1.01	3.37 ± 1.65
NEO	0.84 ± 0.66	1.19 ± 1.14	1.42 ± 1.70	2.52 ± 1.47	2.27 ± 2.25
(KAN-HSA)_complex_	2.22 ± 0.70	5.00 ± 1.10	6.80 ± 1.20	9.00 ± 1.75	10.88 ± 1.64
(NEO-HSA)_complex_	2.83 ± 0.60	4.99 ± 1.05	6.36 ± 0.77	8.04 ± 0.71	10.24 ± 0.99 ^(a)^
One-way ANOVA for repeated measures	F_8_ = 3.61 *p* = 0.00				

Tuckey`s post hoc test: statistically significant difference between ^(a)^ HSA vs. (NEO-HSA)_complex_ for 60 min

*ABTS* assay; *SD standard deviation; *n* = 4–6

**Table 9:** Expected versus designated values of antioxidant activity (Ascorbic Acid Equivalent Antioxidant Capacity, AAEAC) of samples (human serum albumin, HSA; kanamycin, KAN; neomycin, NEO); DPPH assay; *SD standard deviation

	Antioxidant activity (AAEAC) ± SD* [µM AA] DPPH assay					
Samples		Time [min]				
		5	15	30	45	60
(KAN-HSA)_complex_	De***	27.73 ± 1.07	30.63 ± 1.11	32.74 ± 7.41	31.59 ± 5.26	33.11 ± 4.79
	Ex***	16.30 ± 2.31	21.09 ± 3.08	N/A**	20.04 ± 3.88	19.84 ± 4.70
(NEO-HSA)_complex_	De***	18.51 ± 2.31	13.44 ± 1.72	15.53 ± 7.16	14.64 ± 6.11	13.37 ± 5.87
	Ex***	N/A**	N/A**	N/A**	N/A**	N/A**

**N/A not applicable, ***Ex expected, *De* designated

**Table 10:** Expected versus designated values of antioxidant activity AAEAC of the samples; *ABTS* assay; *N/A* not applicable, *Ex* expected, *De* designated, *ad* an additive effect: expected and designated values reveal differences lower than 5%, *s* a synergistic effect: designated values are more than 5% lower for AAEAC when compared with expected values, an—an antagonistic effect: designated values are more than 5% higher for AAEAC when compared with expected values [14]

	Antioxidant activity (AAEAC) ± SD* [µM AA] ABTS assay					
Samples		Time [min]				
		5	15	30	45	60
(KAN-HSA)_complex_	De	2.22 ± 0.70	5.00 ± 1.10	6.80 ± 1.20	9.00 ± 1.75	10.88 ± 1.64
	Ex	1.63 ± 0.60	3.69 ± 0.51	4.73 ± 0.42	5.69 ± 0.54	7.37 ± 1.20
	Effect	s	s	s	s	s
(NEO-HSA)_complex_	De	2.83 ± 0.60	4.99 ± 1.05	6.36 ± 0.77	8.04 ± 0.71	10.24 ± 0.99
	Ex	1.63 ± 0.57	3.74 ± 0.66	4.57 ± 0.41	5.71 ± 0.69	7.00 ± 0.77
	Effect	s	s	s	s	s

*SD standard deviation

Based on the obtained data collected in Table 7, it can be concluded that both HSA and KAN showed antioxidant activity against the DPPH radical. However, it was demonstrated that the antioxidant activity of the (NEO-HSA)_complex_ was statistically significantly lower than (KAN-HSA)_complex_ (p value for (KAN-HSA)_complex_ vs. (NEO-HSA)_complex_ in Tukey’s post hoc test 0.00, 0.00, 0.00, 0.00 for 15, 30, 45, 60 min, respectively). According to the data presented in Table 8, the antioxidant activity of both KAN and NEO against the ABTS cation radical was very low (AAEAC values close to zero). Nevertheless, in the case of KAN, the antioxidant activity was different from zero, regardless of the incubation time. In comparison, the antioxidant activity of HSA was notably higher than that of NEO and KAN. The observed AAEAC values of the ligand–protein complexes were high and did not differ from each other [p value for (KAN-HSA)_complex_ vs. (NEO-HSA)_complex_ in Tuckey`s post hoc test 1.00, 1.00, 1.00, 1.00, 1.00 for 5, 15, 30, 45, 60 min, respectively]. This phenomenon suggests that the effect of both ligands on the antioxidant activity of HSA was very similar (Table 8).

The obtained data indicate that under denaturing conditions, the presence of KAN significantly increases the antioxidant potential of HSA, particularly when compared to the expected results (Table 9). The comparison of the antioxidant activity of the AAEAC values obtained for drug-protein mixtures with the expected values in non-denaturing conditions clearly showed that both KAN and NEO, as a result of interaction with HSA, cause a synergistic effect (Table 10).

## Discussion

This research presented spectroscopic and microcalorimetric techniques to study the antioxidant activity of kanamycin (KAN) and neomycin (NEO) and their effect on the antioxidant potential of human serum albumin (HSA). In addition, the analysis of HSA interaction with ligands was performed.

### Spectrophotometric and spectropolarimetric analysis of the effect of interaction between HSA and ligands

Circular dichroism (CD) is a technique that can be used to evaluate the protein's secondary structure, folding, and binding properties. α-Helix is characterized by minima at 193 nm, 208 nm, and 222 nm. Similarly to our previous studies [15, 16, 35], in the present work, we observed dual minima at wavelengths 208.8 nm and 221.4 nm regardless of ligands (KAN, NEO) presence, at the range between 200 and 250 nm, (Fig. 2, Table 2). This indicates that both KAN and NEO do not modify the α-helical structure of HSA. As presented in Table 3, the percentage of the different types of HSA secondary structures, such as “*α*-helix”, “*β*-sheet”, “turn”, and “other”, did not change. In addition, KAN and NEO do not influence on HSA secondary structure. Using the same technique of CD spectrometry to study the interaction between antibiotics, e.g., kanamycin, and bovine serum albumin (BSA), Judy et al. found that due to the stronger affinity toward BSA (relative to HSA), KAN does not modulate the secondary structure of BSA (statistically non-significant) [36]. Owczarzy et al. [33, 37] analyzed the interaction between 5-alkyl-12(H)-quino[3,4-b][1,4]benzothiazinium derivatives (Salt1, Salt2) and human carrier proteins, including HSA. They observed that Salt1 and Salt2 change the secondary structure of HSA at less than 1% of HSA α-helix or β-sheet content. However, the lack of significant changes in the percentage of α-helix structure is probably due to the used ligand:HSA molar ratio (Table 3). According to Raza et al., an increase in the molar ratio of tobramycin to HSA 6:1 causes a significant decrease in the percentage content of the HSA α-helix structure [38].

The comparison of absorption spectra of a mixture and its components is a simple and widely used method of preliminary detection of potential interactions between each element. Based on the UV–Vis spectra of resveratrol and trypsin, Ren et al. analyzed not only a complex formation of these two substances but also the ligand effect on the protein conformation [39]. As presented in Fig. 3 and Table 4, we detected differences in absorbance values between HSA, KAN, NEO, and binary (KAN-HSA)_complex_ and (NEO-HSA)_complex_ systems. Based on this observation, it can be concluded that HSA interacts with kanamycin and neomycin. To obtain a more detailed analysis, we studied the second derivative of differential absorption spectra (Fig. 4). The wavelengths range from 250 to 270 nm and from 270 to 290 nm allowed us to determine the changes in the environment of phenylalanyl amino acid residues and tyrosyl residues as well as one tryptophanyl residue, respectively [32–34]. The results presented in Table 5 show that KAN and NEO interact with HSA in the region of tryptophanyl and tyrosyl amino acid residues. Additionally, KAN also interacts with phenylalanyl amino acid residues. Due to these differences, further studies are needed to establish and characterize the interaction between KAN and NEO with HSA.

### Calorimetric analysis of HSA interaction with KAN and NEO

As shown in Figs. 5 and 6, respectively the association constants of KAN-HSA and NEO-HSA complexes obtained from nanoITC measurements were relatively low (3.35 ± 0.19) × 10^5^ dm^3^/mol for (KAN-HSA)_complex_ and (9.57 ± 0.22) × 10^4^ dm^3^/mol for (NEO-HSA)_complex_. However, it can be assumed that HSA may not be the most important carrier protein for these two antibiotics. The obtained results are different from those obtained by Sharifi et al. [40]. Using SPR, the authors of the study showed that the K_d_ value of the interaction between NEO and BSA was 2.21 × 10^–5^ M, while K_a_ was around 4.5 × 10^4^ M^−1^. However, conclusions provided by Keswani et al. [41] were different from those reached in the present study. Based on the calorimetric analysis of the NEO-HSA complex, they obtained the exoenergetic reaction (*ΔH* < 0) with the association constant equaled to 1.18 × 10^3^ M^−1^. The authors explained this phenomenon by the use of a non-optimal, too-narrow range of the ligand (i.e., at protein-to-molar ratio NEO:HSA ~ 0.2:1 ÷  ~ 3.75:1). During the ITC method development, the concentrations of the interacting reagents are chosen, so that the resulting isotherm has a fully sigmoidal shape. As demonstrated by our data presented in Figs. 5 and 6, a fully sigmoidal shape of isotherm was possible to obtain using a more widely applicable ranges of molar ratios.

Based on the data presented in Table 6, the values of *K*_*a*_ indicate low affinity of all studied binding sites. The obtained results are in accordance with other scientific works. Using ITC, Kandeel et al. analyzed the binding of streptomycin to BSA. This antibiotic showed very low binding affinity for BSA (the K_a_ value of the order between 10^4^ and 10^3^ M^−1^) and the number of binding sites on the BSA surface (stoichiometry/N) has been identified as 11 ± 0.7 [42]. On the other hand, Scholtan et al. estimated the K_a_ and the n parameter for the binding of streptomycin with HSA equaled 3 × 10^2^ M^−1^ and about 17, respectively [43]. Based on these, it can be concluded that the binding affinity of aminoglycoside antibiotics to albumin is low and the primary binding sites are probably located on the surface of this protein.

The binding reactions of both KAN and NEO with HSA were spontaneous (*ΔG* < 0, Table 6). Formation of (KAN-HSA)_complex_ has an exoenergetic character (*ΔH* < 0, Table 6, Fig. 5), while for (NEO-HSA)_complex_, positive value of ΔH (*ΔH* > 0, Table 6) was obtained and the shape of isotherm (Fig. 6) indicates the endothermic nature of the process.

The comparison of enthalpy (ΔH) and entropy (ΔS) changes allows us to characterize the type of the interaction. When values of enthalpy change are negative (*ΔH* < 0) or close to zero (*ΔH* ~ 0) and, at the same time, the entropy change is positive (*ΔS* > 0), the ionic bonds between the ligand and the protein are predominant. Positive values of both enthalpy change and entropy change (*ΔH* > 0, ΔS > 0) mean that hydrophobic interaction has a dominating role. However, negative values of both enthalpy and entropy change (*ΔH* < 0, *ΔS* < 0) suggest that hydrogen bonds and van der Waals interaction play a key role in protein–ligand stability [41, 44–47]. Based on this, it can be concluded that not only the ionic bonds play the main role in (KAN-HSA)_complex_ but also hydrophobic interaction. This phenomenon occurs due to the value of enthalpy change less than zero, but in a very small range (with simultaneous positive values of entropy change; *ΔH* < 0, *ΔS* > 0). As a result, the NEO-HSA_complex_ is stabilized by hydrophobic interaction (*ΔH* > 0, *ΔS* > 0).

### Spectroscopic analysis of HSA, KAN, NEO, and their mixtures ability to scavenge free radicals in denaturing (DPPH assay) and non-denaturing (ABTS assay) conditions

The main aim of our experiment was to analyze the ability of KAN, NEO, and HSA to regulate and scavenge free radicals. To the best of our knowledge, there is no research evidence confirming the antioxidant capacity of NEO and KAN; therefore, our study has a novel character. Wrześniok et al. showed that KAN modulates the dynamics of antioxidant enzymes in human melanocytes (HEMa-LP) by increasing the activity of catalase (CAT) and superoxide dismutase (SOD) and decreasing the activity of glutathione peroxidase (GPx) [48]. Gibaja et al. studied the influence of kanamycin and cisplatin on Sod1, Gpx1, and Cat genes transcriptional expression using RT-qPCR [49]. Their results showed statistically significantly reduced expression of the Gpx1 gene in the presence of KAN, but the transcript levels of other genes, such as Sod1 and Cat, have not been changed. Current literature results suggest that KAN can effectively modulate enzymatic antioxidant mechanisms, affecting both their synthesis pathways and antioxidant functions [48, 49]. Therefore, we decided to examine the effect of KAN on the antioxidant properties of the non-enzymatic intrinsic factor—HSA.

In addition, we examined the effect of NEO on the endogenous antioxidant mechanisms. Hashemnia et al. showed that NEO at different concentrations affected the activity and properties of catalase (CAT) at 0.5 mg/ml concentration. NEO concentrations below 5.2 mM resulted in increased protection against thermal aggregation, better enzymatic activity, and an increase in the percentage of CAT secondary structure. Moreover, the higher antibiotic concentrations (above 5.2 mM) indicated a decrease in CAT enzymatic activity and increased its sensitivity to thermal aggregation [50].

In the present study, a ligand:protein 2:1 molar ratio was chosen to ensure that binding had no effect on the antioxidant activity of HSA. All tested compounds show antioxidant activity (Tables 7, 8). Moreover, the synergistic effect in the binding of ligands (KAN, NEO) by HSA is observed (Tables 9, 10). Our previous studies have demonstrated that various therapeutic substances modulate the antioxidant activity of HSA [15–17]. A synergistic drug–HSA interaction effect (using the ABTS assay) was observed in the study of naproxen, ketoprofen, quinine, furosemide, and losartan binding to HSA, while an additive interaction effect was observed for diclofenac which is known to induce antioxidant activity [15–17]. Based on the presented data (Tables 7, 8, 9, 10) and our previous studies [15–17], it can be assumed that despite low antioxidant activity (Tables 7, 8), KAN and NEO may have been considered as effective stimulators of HSA antioxidant potential. These drugs contribute to achieving an appropriate level of free radicals in the blood. Simultaneously, due to their low antioxidant potential (Tables 7, 8), they maintain a higher level of free radicals in the target site (e.g., tissues infected by bacteria). The influence of various substances on albumin antioxidant activity has also been studied by other authors [10, 12]. Cao et al. [10] showed that the antioxidant activity of polyphenols against the DPPH radical was higher in the presence of BSA [12]. Han et al. [12] analyzed astilbin (dihydro-flavonol glycoside) binding to HSA [10] and determined that the analyzed ligand affected the secondary structure of the protein. The binding contributed to a decrease in α helix structure in the HSA molecule by approx. 3% and showed a strong interaction with HSA. The K_a_ value for the astilbin–HSA complex was equal to 2.27 × 10^5^ M^−1^ and was found to be antagonistic (as indicated by the DPPH assay). Astilbin has lower antioxidant activity in the complex with HSA than in the absence of protein. The aforementioned study by Han et al. [12] prove that the ability to form a ligand–protein complex affects not only the antioxidant potential of the ligand but also the structure and function of the protein. The synergistic effect of drug–protein interaction should not be attributed only to the simultaneous and cumulative effect of ligand and albumin on free radical quenching. Arcanjo et al. provided evidence indicating the protective role of resveratrol against HSA oxidative damages [51]. HSA's increased resistance to radical damage was not only due to the resveratrol’s antioxidant potential but also as a result of ligand–HSA interaction. Takić et al. [52] demonstrated that polyphenolic compounds with HSA complexes increase the reactivity of the thiol group up to approx. 30% and this is in accordance with the discussion above. Furthermore, this compound, even if negligible or non-existent radical-scavenging capacities (similar to KAN and NEO) exist, might initiate the modulation in the accessibility and/or the chemical reactivity of the Cys-34 thiol moiety (HSA-SH) upon binding with HSA.

## Conclusions

The main aim of the study was to analyze the antioxidant potential of aminoglycoside antibiotics such as kanamycin (KAN) and neomycin (NEO) as well as their influence on human serum albumin potential. Moreover the interaction of KAN and NEO with HSA has been studied.

Using spectroscopic and microcalorimetric techniques, we indicated that KAN and NEO display no antioxidant properties. However, a synergistic effect was discovered between these ligands and HSA. We also found that both KAN and NEO did not cause a noticeable impact on the secondary structure of HSA and demonstrated weak binding affinities to HSA stabilized by hydrophobic and/or ionic interaction. Overall, we assume that the interaction between HSA and aminoglycoside minimizes the harmful consequences of free radicals in the physiological environment. Thus, enhancing the antioxidant abilities of endogenous physiological factors (such as HSA, as well as other antiradical proteins, for example, catalase or superoxide dismutase) may offer new perspectives on pharmacotherapy. Moreover, our studies bring evidence on KAN and NEO potential influence on the homeostasis of free radical concentrations within human physiological systems and antioxidation human defense mechanisms modulation. This can help improve the patient's overall health and return to physiological homeostasis more quickly. Based on this research, our strategy includes in the future in vitro cell testing.

## Data Availability

The data presented in this study are available upon request from the corresponding author.

## Abbreviations

AA: Ascorbic acid
AAEAC: Ascorbic acid equivalent antioxidant capacity
ABTS: 2,2′-Azino-bis(3-ethylbenzothiazoline-6-sulfonic acid)
ad: Additive effect
an: Antagonistic effect
BSA: Bovine serum albumin
CAT: Catalase
CD: Circular dichroism
Cys: Cysteine
De: Designated
DPPH: 2,2-Diphenyl-1-picrylhydrazyl
Eq.: Equation
Ex: Expected
Gpx1: Glutathione peroxidase
HSA: Human serum albumin
ITC: Isothermal titration calorimetry
KAN: Kanamycin
Me: Median
Met: Methionine
N/A: Not applicable
N/S: No signal
NEO: Neomycin
S: Synergistic effect
SD: Standard deviation
SOD: Superoxide dismutase
TOB: Tobramycin
UV: Ultraviolet

## References

[1] Hajam YA, Rani R, Ganie SY, Sheikh TA, Javaid D, Qadri SS (2022). Oxidative stress in human pathology and aging: molecular mechanisms and perspectives. Cells.

[2] Chand K, Rajeshwari HA, Singh M, Santos MA, Keri RS (2017). A review on antioxidant potential of bioactive heterocycle benzofuran: natural and synthetic derivatives. Pharmacol Rep.

[3] Di Meo S, Venditti P (2020). Evolution of the knowledge of free radicals and other oxidants. Oxid Med Cell Longev.

[4] Elias RJ, Kellerby SS, Decker EA (2008). Antioxidant activity of proteins and peptides. Crit Rev Food Sci Nutr.

[5] Mirończuk-Chodakowska I, Witkowska AM, Zujko ME (2018). Endogenous non-enzymatic antioxidants in the human body. Adv Med Sci.

[6] Carter DC, He XM (1990). Structure of human serum albumin. Science.

[7] Rabbani G, Ahn SN (2019). Structure, enzymatic activities, glycation and therapeutic potential of human serum albumin: a natural cargo. Int J Biol Macromol.

[8] Maciążek-Jurczyk M (2014). Phenylbutazone and ketoprofen binding to serum albumin. Fluoresc Study Pharmacol Rep.

[9] Anraku M, Chuang VT, Maruyama T, Otagiri M (2013). Redox properties of serum albumin. Biochim Biophys Acta.

[10] Han X, Sun J, Niu T, Mao B, Gao S, Zhao P (2022). Molecular insight into the binding of astilbin with human serum albumin and its effect on antioxidant characteristics of astilbin. Molecules.

[11] De Simone G, di Masi A, Ascenzi P (2021). Serum albumin: a multifaced enzyme. Int J Mol Sci.

[12] Cao H, Chen X, Yamamoto K (2012). Bovine serum albumin significantly improves the DPPH free radical scavenging potential of dietary polyphenols and gallic acids. Anticancer Agents Med Chem.

[13] Olszowy-Tomczyk M (2020). Synergistic, antagonistic and additive antioxidant effects in the binary mixtures. Phytochem Rev.

[14] Guimarães R, Barros L, Carvalho AM, Ferreira IC (2011). Infusions and decoctions of mixed herbs used in folk medicine: Synergism in antioxidant potential. Phytother Res.

[15] Rogóż W, Pożycka J, Kulig K, Owczarzy A, Szkudlarek A, Maciążek-Jurczyk M (2023). New look at the metabolism of nonsteroidal anti-inflammatory drugs: influence on human serum albumin antioxidant activity. J Biomol Struct Dyn.

[16] Rogóż W, Pożycka J, Owczarzy A, Kulig K, Maciążek-Jurczyk M (2022). Comparison of losartan and furosemide interaction with HSA and their influence on HSA antioxidant potential. Pharmaceuticals (Basel).

[17] Rogóż W, Lemańska O, Pożycka J, Owczarzy A, Kulig K, Muhammetoglu T (2022). Spectroscopic analysis of an antimalarial drug's (quinine) influence on human serum albumin reduction and antioxidant potential. Molecules.

[18] Krause KM, Serio AW, Kane TR, Connolly LE (2016). Aminoglycosides: an overview. Cold Spring Harb Perspect Med.

[19] Dezanet C, Kempf J, Mingeot-Leclercq MP, Décout JL (2020). Amphiphilic aminoglycosides as medicinal agents. Int J Mol Sci.

[20] Yao CJ, Li YL, Pu MJ, Luo LH, Xiong Q, Xie FJ (2021). Aminoglycosides with anti-MRSA activity: a concise review. Curr Top Med Chem.

[21] Eyler RF, Shvets K (2019). Clinical pharmacology of antibiotics. Clin J Am Soc Nephrol.

[22] Jospe-Kaufman M, Siomin L, Fridman M (2020). The relationship between the structure and toxicity of aminoglycoside antibiotics. Bioorg Med Chem Lett.

[23] Venyaminov SY, Vassilenko KS (1994). Determination of protein tertiary structure class from circular dichroism spectra. Anal Biochem.

[24] Sreerama N, Woody RW (2000). Estimation of protein secondary structure from circular dichroism spectra: comparison of CONTIN, SELCON, and CDSSTR methods with an expanded reference set. Anal Biochem.

[25] Bou-Abdallah F, Sprague SE, Smith BM, Giffune TR (2016). Binding thermodynamics of diclofenac and naproxen with human and bovine serum albumins: a calorimetric and spectroscopic study. J Chem Thermodyn.

[26] Kaspchak E, Goedert AC, Igarashi-Mafra L, Mafra MR (2019). Effect of divalent cations on bovine serum albumin (BSA) and tannic acid interaction and its influence on turbidity and in vitro protein digestibility. Int J Biol Macromol.

[27] Mic M, Pîrnău A, Floare CG, Bogdan M (2020). Study of the binding affinity between imatinib and α-1 glycoprotein using nuclear spin relaxation and isothermal titration calorimetry. Int J Biol Macromol.

[28] Ràfols C, Amézqueta S, Fuguet E, Bosch E (2018). Molecular interactions between warfarin and human (HSA) or bovine (BSA) serum albumin evaluated by isothermal titration calorimetry (ITC), fluorescence spectrometry (FS) and frontal analysis capillary electrophoresis (FA/CE). J Pharm Biomed Anal.

[29] Yuan L, Liu M, Shi Y, Yan H, Han J, Liu L (2019). Effect of (-)-epicatechin-3-gallate and (-)-epigallocatechin-3-gallate on the binding of tegafur to human serum albumin as determined by spectroscopy, isothermal titration calorimetry, and molecular docking. J Biomol Struct Dyn.

[30] Al-Salahi R, Taie HAA, Bakheit AH, Marzouk M, Almehizia AA, Herqash R (2019). Antioxidant activities and molecular docking of 2-thioxobenzo[g]quinazoline derivatives. Pharmacol Rep.

[31] Swain S, Rautray TR (2021). Estimation of trace elements, antioxidants, and antibacterial agents of regularly consumed indian medicinal plants. Biol Trace Elem Res.

[32] Ichikawa T, Terada H (1979). Estimation of State amount of Phenylalanine residues in proteins by second derivative spectrophotometry. Biochim Biophys Acta.

[33] Owczarzy A, Zięba A, Pożycka J, Kulig K, Rogóż W, Szkudlarek A (2021). Spectroscopic studies of quinobenzothiazine derivative in terms of the in vitro interaction with selected human plasma proteins Part 1. Molecules.

[34] Terada H, Inoue Y, Ichikawa T (1984). Second derivative spectral properties of tryptophan and tyrosine residues in proteins. Effects of guanidine hydrochloride and dodecyl sulfate in the residues in lysozyme, ribonuclease and serum albumin. Chem Pharm Bull.

[35] Maciążek-Jurczyk M, Morak-Młodawska B, Jeleń M, Kopeć W, Szkudlarek A, Owczarzy A (2021). The Influence of oxidative stress on serum albumin structure as a carrier of selected diazaphenothiazine with potential anticancer activity. Pharmaceuticals.

[36] Judy E, Kishore N (2021). Correlating the properties of antibiotics with the energetics of partitioning in colloidal self-assemblies and the effect on the binding of a released drug with a target protein. Langmuir.

[37] Owczarzy A, Rogóż W, Kulig K, Pożycka J, Zięba A, Maciążek-Jurczyk M (2023). Spectroscopic studies of quinobenzothiazine derivative in terms of the in vitro interaction with selected human plasma proteins: Part 2. Molecules.

[38] Raza M, Wei Y, Jiang Y, Ahmad A, Raza S, Ullah S (2017). Molecular mechanism of tobramycin with human serum albumin for probing binding interactions: multi-spectroscopic and computational approaches. New J Chem.

[39] Ren G, Sun H, Guo J, Fan J, Li G, Xu S (2019). Molecular mechanism of the interaction between resveratrol and trypsin via spectroscopy and molecular docking. Food Funct.

[40] Sharifi M, Ezzati Nazhad Dolatabadi J, Fathi F, Zakariazadeh M, Barzegar A, Rashidi M (2017). Surface plasmon resonance and molecular docking studies of bovine serum albumin interaction with neomycin: kinetic and thermodynamic analysis. Bioimpacts.

[41] Keswani N, Choudhary S, Kishore N (2010). Interaction of weakly bound antibiotics neomycin and lincomycin with bovine and human serum albumin: biophysical approach. J Biochem.

[42] Kandeel M, Nakashima R, Kitamura Y, Balaha M, Abdelaziz M, Kitade Y (2013). Thermodynamics and molecular bases of the interaction of ampicillin and streptomycin at their binding sites of bovine serum albumin. J Therm Anal Calorim.

[43] Scholtan W, Rosenkranz H (1978). Ionic binding of aminoglycosides to human serum albumin in the absence of divalent cations IV Effect of structure, pH and concentration. Infection.

[44] Aki H, Yamamoto M (1994). Thermodynamic characterization of drug binding to human serum albumin by isothermal titration microcalorimetry. J Pharm Sci.

[45] Ross D, Subramanian S (1981). Thermodynamics of protein association reactions: forces contributing to stability. Biochemistry.

[46] Marković OS, Cvijetić IN, Zlatović MV, Opsenica IM, Konstantinović JM, Terzić Jovanović NV (2018). Human serum albumin binding of certain antimalarials. Spectrochim Acta A.

[47] He Y, Zhou C, Li C, Zhou G (2021). Effect of incubation temperature on the binding capacity of flavor compounds to myosin. Food Chem.

[48] Wrześniok D, Otręba M, Beberok A, Buszman E (2013). Impact of kanamycin on melanogenesis and antioxidant enzymes activity in melanocytes–an in vitro study. J Cell Biochem.

[49] Gibaja A, Alvarado JC, Scheper V, Carles L, Juiz JM (2022). Kanamycin and cisplatin ototoxicity: differences in patterns of oxidative stress, antioxidant enzyme expression and hair cell loss in the Cochlea. Antioxidants (Basel).

[50] Hashemnia S, Mokhtari Z, Tashkhourian J, Moosavi-Movahedi AA (2015). Effect of covalent attachment of neomycin on conformational and aggregation properties of catalase. Indian J Biochem Biophys.

[51] Arcanjo NMO, Luna C, Madruga MS, Estévez M (2018). Antioxidant and pro-oxidant actions of resveratrol on human serum albumin in the presence of toxic diabetes metabolites: Glyoxal and methyl-glyoxal. Biochim Biophys Acta.

[52] Takić MM, Jovanović VB, Pavićević ID, Uzelac TN, Aćimović JM, Ristić-Medić DK (2016). Binding of enterolactone and enterodiol to human serum albumin: increase of cysteine-34 thiol group reactivity. Food Funct.

